# Predictors of Reading Comprehension in Children With Cochlear Implants

**DOI:** 10.3389/fpsyg.2019.02155

**Published:** 2019-09-24

**Authors:** Malin Wass, Lena Anmyr, Björn Lyxell, Elisabet Östlund, Eva Karltorp, Ulrika Löfkvist

**Affiliations:** ^1^Department of Business Administration, Technology and Social Sciences, Luleå University of Technology, Luleå, Sweden; ^2^Department of Clinical Science, Intervention and Technology, Karolinska Institutet, Stockholm, Sweden; ^3^Department of Social Work in Health, Karolinska University Hospital, Stockholm, Sweden; ^4^Department of Special Needs Education, University of Oslo, Oslo, Norway; ^5^Department of Behavioural Sciences and Learning, Linköping University, Linköping, Sweden; ^6^Department of Otorhinolaryngology, Karolinska University Hospital, Stockholm, Sweden

**Keywords:** reading comprehension, children with CI, vocabulary, word decoding, cochlear implants, simple view of reading, lexical quality hypothesis

## Abstract

Children with a profound hearing loss who have been implanted with cochlear implants (CI), vary in terms of their language and reading skills. Some of these children have strong language skills and are proficient readers whereas others struggle with language and both the decoding and comprehension aspects of reading. Reading comprehension is dependent on a number of skills where decoding, spoken language comprehension and receptive vocabulary have been found to be the strongest predictors of performance. Children with CI have generally been found to perform more poorly than typically hearing peers on most predictors of reading comprehension including word decoding, vocabulary and spoken language comprehension, as well as working memory. The purpose of the current study was to investigate the relationships between reading comprehension and a number of predictor variables in a sample of twenty-nine 11–12-year-old children with profound hearing loss, fitted with CI. We were particularly interested in the extent to which reading comprehension in children with CI at this age is dependent on decoding and receptive vocabulary. The predictor variables that we set out to study were word decoding, receptive vocabulary, phonological skills, and working memory. A second purpose was to explore the relationships between reading comprehension and demographic factors, i.e., parental education, speech perception and age of implantation. The results from these 29 children indicate that receptive vocabulary is the most influential predictor of reading comprehension in this group of children although phonological decoding is, of course, fundamental.

## Introduction

Children with a profound hearing loss who have been implanted with cochlear implants (CI), show substantial variation in reading skills. Some children have been reported to read well within the normal range of hearing peers on measures of word decoding and reading comprehension (e.g., Dillon et al., 2011). Many others, however, struggle with both the decoding and comprehension aspects of reading (Geers, 2003; Kyle and Harris, 2006; Harris and Terlektsi, 2010; Geers and Hayes, 2011).

Some previous research has been focused on the causes of variation in reading ability within this group of children (e.g., Connor and Zwolan, 2004; Dillon et al., 2011; Von Muenster and Baker, 2014) but the use of different predictor variables between studies as well as the heterogeneity of the children included in the research makes it difficult to draw general conclusions. Examples of the variation that can be seen across studies, are the children’s age range, main communication mode, and the predictors that have been measured.

This study set out to investigate the cognitive and linguistic predictors that are known to be most relevant for reading comprehension in children with typical hearing, in a group of 11–12-year-old children with profound hearing loss who use CI. This is in contrast to previous studies on reading comprehension in children with CI which have mostly been focused on demographic factors (c.f. Connor and Zwolan, 2004; Dillon et al., 2011) and/or have included children with broad age ranges. The children included in this research used mainly oral communication and the majority of them were bilaterally implanted.

The theoretical background of reading comprehension and its main cognitive and linguistic predictors, as documented in typically hearing children, is reviewed below, followed by a summary of findings from previous research on children with CI.

### Reading Comprehension in Children With Typical Hearing

One of the most fundamental prerequisites for reading comprehension is the ability to efficiently decode written words. Early reading typically involves the effortful grapheme-phoneme conversion by which children sound words out by adding and blending letter sounds (Coltheart et al., 2001). Word decoding then gradually becomes more automatized and effortless as whole words are recognized instantly by sight, so called *orthographic word recognition* (Ehri, 2005, 2014). Thereby, more cognitive resources can be used for comprehension and the acquisition of new information from the text (Perfetti, 2007).

In addition to word decoding, the reader further needs language skills that enable him or her to understand what is being read. The relative importance of decoding and language skills for reading comprehension has been found to vary depending on the children’s age (e.g., Ouellette and Beers, 2010; Melby-Lervåg and Lervåg, 2014). That is, decoding is relatively more important in the early stages of reading development whereas language and vocabulary generally plays a greater role for children who have learned to master basic word reading skills (e.g., Lervåg and Aukrust, 2010; Melby-Lervåg and Lervåg, 2014). The nature of the language skills that are most relevant for reading comprehension is explained differently in two models of reading comprehension; *the Simple View of Reading* (Gough and Tunmer, 1986; Tunmer and Chapman, 2012) and the *Lexical Quality Hypothesis* (Perfetti, 2007).

The Simple View of Reading (SVR) suggests that reading comprehension is constituted by two components: word decoding and comprehension of oral language and that both components are equally important (Gough and Tunmer, 1986; Tunmer and Chapman, 2012). According to Tunmer and Chapman (2012), language comprehension is a hypothetical construct, which can be split up into component processes such as the retrieval of individual words in lexical memory (receptive vocabulary) and the knowledge about how words and syntactic structures should be used. The broad definition of language comprehension in the SVR makes it difficult to measure with precision (e.g., Ouellette and Beers, 2010) and an increasing number of correlational studies suggest that larger proportions of reading comprehension are explained by variance in vocabulary than by listening comprehension (Braze et al., 2007; Protopapas et al., 2007; Verhoeven and Van Leeuwe, 2008; Ouellette and Beers, 2010; Olson et al., 2011), in particular for children beyond the earliest stages of reading development.

The lexical quality hypothesis (LQH, Perfetti, 2007; Perfetti and Stafura, 2014), on the other hand, stresses the importance of word knowledge and assumes that it, together with decoding ability, is the most central component of reading comprehension. According to the LQH, the quality of word representations within any reader’s vocabulary, varies depending on how familiar the reader is with the word in terms of several aspects including lexical meaning, pragmatic use, and orthographic and phonological characteristics (Perfetti, 2007). The LQH assumes that knowledge of word meaning affects reading comprehension not only indirectly via its effect on listening comprehension but also directly. This view is supported by results from hierarchical regression analyses which show that vocabulary significantly contributes to reading comprehension beyond the effects of language comprehension (Ouellette and Beers, 2010; Perfetti and Stafura, 2014).

Irrespective of the theoretical framework applied in research, there has been some confusion regarding the definition of the decoding component in reading comprehension, whether it refers to phonological decoding, orthographic word recognition or both (Ouellette and Beers, 2010; Tunmer and Chapman, 2012). According to Tunmer and Greaney (2010) the most sensitive measures of decoding should be expected to vary depending on children’s level of reading development. That is, for beginning readers, phonological decoding is the most frequently used decoding strategy, which should be used as the main measure of decoding whereas word recognition or even speed of word recognition should be used as more sensitive measures of decoding for advanced readers. According to Tunmer and Chapman (2012), a composite measure of both phonological decoding and orthographic word recognition is suitable for assessment of decoding skill for a broad range of readers.

Other cognitive skills that predict additional variance in children’s reading comprehension include working memory (e.g., Currie and Cain, 2015), a variable which is relatively more important for longer passages of text, and phonological skills (Melby-Lervåg and Lervåg, 2014) which is generally more important in early stages of reading.

### Reading Comprehension in Children With CI

When it comes to the general cognitive and linguistic predictors of reading comprehension, children with CI have typically been found to perform more poorly than hearing peers on both decoding (Geers, 2003; Geers and Hayes, 2011; Nakeva von Mentzer et al., 2014), vocabulary (Geers et al., 2009; Fagan and Pisoni, 2010; Dillon et al., 2011; Coppens et al., 2013; Walker et al., 2019) and spoken language comprehension (e.g., Geers et al., 2009), as well as phonological and complex working memory (e.g., Wass et al., 2008). This would, in turn, suggest generally poorer preconditions for reading comprehension in this group of children.

A few studies have specifically investigated the relationships between reading comprehension and various predictor variables in children with CI (e.g., Connor and Zwolan, 2004; Asker-Árnason et al., 2007; Vermeulen et al., 2007; Von Muenster and Baker, 2014). The age range of the participating children is, however, typically relatively broad and the measures used to assess reading and predictors of reading vary substantially between studies.

Connor and Zwolan (2004) explored a number of demographic, cognitive and linguistic predictors of reading comprehension in ninety-one 11 year-old children with CI. They found age at implantation to have strong effects on reading comprehension (the younger the better) both directly and through its positive effects on vocabulary growth. It should be noted here that the children included in their study were implanted at 6.7 years of age on average and thus got access to oral language relatively late. This is because prelingually deaf children, who have been implanted later than 3.5 years of age, have been shown to benefit less from cochlear implantation and typically show poorer development of speech and comprehension of oral language (Kral and Sharma, 2012). The study by Connor and Zwolan did not include a measure of word decoding and thus the relative effects of decoding and oral language cannot be compared.

The children studied by Dillon et al. (2011) were implanted relatively earlier, at 2.5 years of age on average, but the age range was broader (6–14 years). Twenty-seven English-speaking children with CI were included in their study. Although there was a substantial individual variation within the group, the children performed on average within the typical range for hearing children on measures of decoding and reading comprehension whereas their receptive vocabulary was below this range. Reading comprehension, as measured by the PIAT- R (Markwardt, 1998) was further found to be strongly associated with receptive vocabulary and phonological awareness. The strength of these correlations were, however, not compared to the correlation between reading comprehension and decoding. The authors note that age at implantation was moderately correlated with non-word reading (*r* = 0.56) and reading comprehension (*r* = 0.43), and duration of implant use was strongly correlated with measures of phonological awareness and reading (*r* = 0.86).

An Australian study by Von Muenster and Baker (2014) on 47 children with unilateral CI aged 5;4–12;6 years, reported strong correlations between reading comprehension, as measured by the Neale Analysis of Reading Ability and each of the following skills: word and non-word decoding, r≈0.8–0.9, expressive and receptive language (r≈0.8). There was also a strong correlation between reading comprehension and receptive vocabulary as measured by PPVT (Dunn and Dunn, 2007), r≈0.7. Notably, none of the measures of reading (decoding and comprehension) used in their study was significantly related to measures of auditory perception, age at implantation or duration of implant use.

Results from a sample of fifty Dutch children with CI in a similar age range (7–16 years) was reported by Vermeulen et al. (2007). The authors found strong correlations between reading comprehension and measures of both word recognition and receptive vocabulary. The latter was, however, a relatively stronger predictor, explaining 29% of the variance in reading comprehension after age and educational factors had been taken into account.

To sum up, the few studies on children with CI which have investigated cognitive and linguistic factors associated with reading comprehension, have typically included children in broad age ranges. Since the predictors of reading comprehension are known to vary with age and level of reading development, it is therefore important to study the theoretically most relevant predictors in children with CI at more narrow age ranges in order to find the most important predictors of successful reading at every particular stage in development. Based on findings from typically hearing children, decoding should be expected to play a greater role for reading comprehension in younger children who may not yet read fluently whereas vocabulary should be relatively more important as children become fluent readers (c.f. Lervåg and Aukrust, 2010). Furthermore, the extent to which age at implantation, decoding and language and vocabulary factors contribute to reading comprehension should be expected to vary depending on the characteristics of the sample studied. For example, age at implantation may be more important for reading (and language) for children who have been implanted relatively late (c.f. Kral and Sharma, 2012).

The purpose of the current study was to investigate the relationships between reading comprehension and a number of cognitive and linguistic predictor variables in a sample of twenty-nine 11–12-year-old children with profound hearing loss, fitted with CI. We were particularly interested in the extent to which reading comprehension in children with CI at this age was dependent on decoding and receptive vocabulary. The predictor variables that we set out to study were word decoding, receptive vocabulary, phonological skills, and working memory.

A second purpose was to explore the relationships between reading comprehension and demographic factors, i.e., parental education, speech perception and age of implantation.

## Materials and Methods

### Participants

Twenty-nine children (14 girls) participated in this study as part of a longitudinal research project on reading development and language in children with CI. Results from an earlier measurement have been reported previously for most of the children included in the current sample (Wass et al., 2019). The inclusion criteria were that all children should be able to follow the regular national school curriculum and perform at or above the 25th percentile on Raven’s Colored Progressive Matrices (Raven et al., 2003).

Written informed consent was obtained from the children and from their parents. The children were, on average, 11;8 (years; months) of age at the time of testing (range: 11;0–12;8).

The mean age at implantation of the first CI was 24 months (range 7–69 months). Twenty-six of the children (90%) had bilateral implants and were implanted with their second CI at 29 months of age on average (range 8–105 months). Three children had bimodal hearing (CI and hearing aid).

Twenty-three of the children had used oral communication only for their whole lives, 3 children had used oral communication in combination with sign support until they started to speak themselves but had exclusively used oral communication since then. Three children were reported to have used oral communication in combination with sign language from the time they were diagnosed with their hearing loss and that they still used both communication modes.

All children were tested at the hearing implant clinic, Karolinska University Hospital at their annual follow-up appointment. They also attended regular speech and listening rehabilitation at their local hospitals during the rest of the year (Wass et al., 2019).

The sample was heterogeneous in terms of cause of deafness and age of implantation of first and second CI. Etiology and age at implantation for the sample are summarized in Table 1.

**TABLE 1:** Age at implantation and etiology.

	**Mean**	**SD (range)**
Age at CI1 (months)	24.0	17.8 (7–69)
Age at CI2 (months)	28.4	21.0 (8–105)
Etiology	#of children	Proportion
Acquired		9/29
Congenital CMV	4	
Meningitis	5	
Genetic		12/29
Unspecific heredity^∗^	2	
Connexin 26	3	
Usher type 1	2	
Jervell-Lange Nielsen syndr.	3	
Pendred’s syndr.	2	
Unknown	8	8/29

*^∗^Close family members also have a hearing impairment.*

Speech perception in quiet as measured by phonetically balanced lists was, on average, 81.1% (SD: 15.9). One child had missing speech perception data. Raven Colored Progressive Matrices test (Raven et al., 2003) was used to measure non-verbal cognitive ability and the participants’ percentile scores ranged between 25 and 95.

### Test Measures

The Swedish reading test LäSt (Elwér et al., 2009) was used to measure decoding of words and non-words, respectively. Reading comprehension was assessed with a Swedish version of the Woodcock Reading Mastery Test (Byrne et al., 2009).

Receptive Vocabulary was measured with Peabody Picture Vocabulary Test (PPVT-III; Dunn and Dunn, 2007).

A Sentence Completion and Recall task (Wass et al., 2008) was used to measure complex working memory. In this task, the children are asked to fill in missing words in sets of sentences e.g., “Crocodiles are green. Tomatoes are …”. After every set of sentences, the child should also repeat back the words that she/he had filled in. The sentence sets consisted of two, three, and four sentences and the total number of correctly stored and reproduced words was recorded by the test leader, with a maximum score of 18.

A phoneme deletion task (Magnusson and Nauclér, 1993) was used to assess phonological skills. In this test, the children are asked to remove phoneme segments of spoken words, e.g., “Say summer without an ‘s’. The maximum score is 12.

### Procedure

The children were individually tested by an experienced speech language pathologist and an audiologist working at the hearing implant clinic. All language and cognitive tests were presented in random order and administered during two consecutive days, in two 1-hour sessions. The audiologist tested the children’s hearing ability during the first day. The test instructions were given in oral language.

### Analyses

Relationships between the various skills were analyzed in correlation and hierarchical regression analyses. We only had comparison data from typically hearing children for the reading comprehension test, for which we had results from 21 children with typical hearing who were 10–11 years of age. The average performance on the reading comprehension test was 31.6 (SD: 6.2) for these children and the reading comprehension performance of the children with CI was compared to this comparison data.

## Results

Means and standard deviations for all test measures are displayed in Table 2 and correlations are displayed in Table 3.

**TABLE 2:** Tests administered; means, standard deviations, and range.

**Ability**	**Test**	**Quantification**	**Mean(SD; range)**	***N***
**Measures of language and cognitive skills**:				
Reading comprehension	Woodcock reading mastery test	Number of correctly completed sentences (maximum score = 68)	35 (6.74; 22–51)	29
Non-word decoding fluency	LäSt	Number of correctly read non-words in 2^∗^45 s (maximum score = 126)	79.9 (20.10; 41–114)	28
Word decoding fluency	LäSt	Number of correctly read non-words in 2^∗^45 s (maximum score = 200)	139.9 (22.1; 105–176)	28
Receptive vocabulary	PPVT-III	Number of correctly identified pictures (maximum score = 228)	152.0 (28.6; 80–194)	29
Complex working memory	Sentence completion and recall	Number of words correctly recalled (maximum score = 18)	13.62 (2.57; 6–17.5)	29
Phonological skills	Phoneme deletion	Number of correct answers (maximum score = 12)	11.72 (0.70; 9–12)	29

*Missing data for one participant on non-word decoding fluency and word decoding fluency due to fatigue.*

**TABLE 3:** Bivariate correlations.

	**1**	**2**	**3**	**4**	**5**	**6**	**7**	**8**	**9**	**10**
1. Age CI 1	1									
2. Speech perception	–0.305	1								
3. Parental education^a^	–0.132	–0.003	1							
4. Receptive vocabulary	–0.204	0.218	0.593^∗∗^	1						
5. Phoneme deletion	0.032	0.265	0.341	0.375^∗^	1					
6. Complex WM	–0.226	0.417^∗^	0.264	0.639^∗∗∗^	0.484^∗∗^	1				
7. nw-decoding	–0.195	0.225	0.435^∗^	0.458^∗^	0.469^∗^	0.393^∗^	1			
8. wd-decoding	–0.134	0.188	0.381^∗^	0.357	0.431^∗^	0.396^∗^	0.815^∗∗∗^	1		
9. Decoding composite average	–0.171	0.216	0.427^∗^	0.425^∗^	0.472^∗^	0.414^∗^	0.948^∗∗∗^	0.957^∗∗∗^	1	
10. Reading comprehension	–0.152	0.069	0.552^∗∗^	0.783^∗∗∗^	0.501^∗∗^	0.447^∗^	0.512^∗∗^	0.417^∗^	0.485^∗∗^	1

*^∗^Correlation is significant at the 0.05 level (2-tailed). ^∗∗^Correlation is significant at the 0.01 level (2-tailed). ^∗∗∗^p < 0.001. ^*a*^Correlation coefficients denote point-biseral correlations, r_*pb*_.*

Twenty-six out of the 29 children with CI (almost 90%) performed within 1 SD from the mean of the comparison group on the reading comprehension measure.

Significant bivariate correlations were found between reading comprehension and all of the cognitive/linguistic predictor variables *with r*s in the moderate-strong range.

Neither age at implantation of first or second CI nor speech perception in quiet at the time of the follow-up visit were significantly correlated with any of the measures of reading.

Parental education was coded as a dichotomous variable, that is children whose parents’ highest education was high-school or a shorter education constituted one group (*N* = 10) and the other group had parents with a university degree (*N* = 19). The effects of parental education on children’s reading ability was explored in a Mann-Whitney group comparison. There were no significant differences between the groups on chronological age or age of implantation of the first CI. The two groups, however, differed significantly on reading comprehension (*U* = 35, *p* < 0.01) and also on non-word decoding (*U* = 46.5, *p* < 0.05). The group difference on the measure of word decoding approached significance (*U* = 50.0, *p* = 0.055). Point-biseral correlations further showed that parental education was significantly correlated with receptive vocabulary, *r*_*pb*_ = 0.593 (*p* < 0.001), non-word decoding, *r*_*pb*_ = 0.435, *p* < 0.05, and word decoding, *r*_*pb*_ = 0.381, *p* < 0.05, and with reading comprehension, r_*pb*_ = 0.552, *p* < 0.01.

Subsequently, a set of hierarchical regression analyses were conducted with reading comprehension as the dependent variable. The results of these analyses are presented in Table 4 and Table 5. Age at testing was not included as a predictor in any of the regression analyses as it was not correlated with reading comprehension (*r* = 0.044).

**TABLE 4:** Significant predictors of reading comprehension.

**Models**	**Variables**	**R**	**R^2^**	**Adj. R^2^**	**ΔR^2^**	**B**	**SE**	**β**	**t**
1	Step 1	0.540	0.292	0.265					
	NVIQ					0.174	0.053	0.540^∗∗^	3.27
2	Step 2	0.621	0.386	0.336	0.094				
	NVIQ					0.134	0.054	0.417^∗^	2.47
	Decoding					0.111	0.057	0.330	1.95
3	Step 3	0.806	0.649	0.605	0.264				
	NVIQ					−036	0.048	0.111	0.744
	Decoding					0.056	0.045	0.168	1.24
	PPVT					0.153	0.036	0.649^∗∗∗^	4.25

*^∗^*p* < 0.05, ^∗∗^*p* < 0.01, ^∗∗∗^*p* < 0.001.*

**TABLE 5:** Significant predictors of reading comprehension.

**Models**	**Variables**	**R**	**R^2^**	**Adj. R^2^**	**ΔR^2^**	**B**	**SE**	**β**	**t**
1	Step 1	0.540	0.292	0.265					
	NVIQ					0.174	0.053	0.540^∗∗^	3.27
2	Step 2	0.640	0.410	0.363	0.118				
	NVIQ					0.132	0.053	0.411^∗^	2.50
	Non-word decoding					0.123	0.055	0.368^∗^	2.24
3	Step 3	0.807	0.652	0.608	0.242				
	NVIQ					0.038	0.047	0.119	0.81
	Non-word decoding					0.060	0.046	0.180	1.32
	PPVT					0.149	0.037	0.633^∗∗^	4.08

*^∗^*p* < 0.05, ^∗∗^*p* < 0.01, ^∗∗∗^*p* < 0.001.*

In the first analysis (displayed in Table 4), Raven’s CPM, a composite measure of decoding (LäSt words + LäSt non-words) and receptive vocabulary (PPVT-III) were used as independent variables. Raven’s percentile was entered at the first step and decoding was entered at the second step. Together these two variables accounted for 38.6 percent of the variance in reading comprehension but the decoding measure did not significantly improve model fit.

When receptive vocabulary was entered at the third step, the model predicted 64.9 percent of the variance in reading comprehension. Neither non-verbal IQ nor decoding contributed significantly to reading comprehension once receptive vocabulary had been entered into the model.

A second set of hierarchical regression analyses were then conducted (Table 5) in which the measure of phonological decoding (LäSt non-words) was used instead of the composite measure of decoding. In this analysis, all three variables, non-verbal IQ, non-word decoding and receptive vocabulary, significantly improved model fit and together they accounted for 65.2 percent of performance on the reading comprehension test although only the beta weight for receptive vocabulary was significant.

In a third set of regression analyses we wanted to explore the effects of phonological awareness, complex working memory and parental education. The contribution of these variables was explored in three separate analyses in which Raven’s CPM was entered at the first step, non-word decoding at the second step and phoneme deletion, sentence completion and recall and parental education, respectively, were entered at the third step. Neither of these variables significantly predicted reading comprehension.

## Discussion

The aim of this research was to explore the predictors of reading comprehension in 11–12-year-old Swedish children with profound hearing loss using CI. The results from these 29 children indicate that receptive vocabulary is the most influential predictor of reading comprehension in this group of children although decoding is still, of course, important.

Although the focus of this research was not to compare the performance of children with CI to typically hearing peers, it should be mentioned that most of the children in our sample (26/29) performed within 1 SD below NH mean or above, on the measure of reading comprehension. These children thus had relatively high performance compared to the approximately 50% of children with CI who have previously been reported to perform within this range (e.g., Geers, 2003; Asker-Árnason et al., 2007). The difference in results may, of course, be due to the size and representativeness of our sample, at least compared to the 181 children included in the study by Geers (2003). The results may also be due to the fact that the children who participated in the study by Geers (2003) were implanted between 1990-1996 whereas the children included in the current sample were all implanted approximately a decade later and the technological advances in implant technology may thus have improved the auditory preconditions for reading development in this population of children. On the other hand, neither age at implantation of first and second CI nor speech perception in quiet at the time of testing were significantly correlated with any of the measures of reading in our sample of children. These findings are in line with results reported by Von Muenster and Baker (2014) who did not find significant relationships between reading and hearing measures. It should be noted that the children included in the current study and the children who participated in the sample by Von Muenster and Baker were implanted at approximately the same age, i.e., slightly above 3 years. It is possible that effects of age at implantation can only be seen for children implanted at a relatively later age (c.f. Connor and Zwolan, 2004).

The results from the hierarchical regression analyses showed that receptive vocabulary was the main predictor of reading comprehension in our sample of children with CI. Interestingly, when a composite measure of word decoding and non-word decoding was used in the analysis, this composite measure of decoding failed to predict a significant proportion of variance in reading comprehension even before receptive vocabulary was added to the model. When the composite measure of decoding was replaced by the non-word decoding measure in the analysis, all three variables significantly predicted model fit although only the beta-weights for receptive vocabulary turned out to be significant. The regression model in which non-word decoding was used as a decoding measure further explained more variance in reading comprehension than the composite measure of word and non-word decoding. This difference was not significant but it is interesting in light of the discussion about what aspects of decoding are most important for children of different ages. There may be a tendency for phonological decoding to be relatively more important for reading comprehension than composite measures of phonological and orthographic decoding for children with CI at age 11–12 years. According to Tunmer and Greaney (2010), phonological decoding is the most frequently used decoding strategy for beginning readers, and according to the current results it seems that phonological decoding is still the most influential aspect of decoding for children with CI at age 11–12.

These findings suggest that, for children with CI at this age, vocabulary is relatively more important for reading comprehension than measures of word decoding. In comparison, recent results from Bell et al. (2019) suggest that decoding is relatively more important than language measures at age 8 whereas the opposite pattern was found in an age-matched comparison group of typically hearing children. It thus seems that at age 11–12, the decoding skills of children with CI has reached the level of decoding skill at which differences in reading comprehension are, similar to typically hearing children, more dependent on vocabulary. The vocabulary knowledge of children with CI should further be expected to vary in part depending on the length of auditory deprivation before cochlear implantation (Fagan and Pisoni, 2010) and in general as children have been both diagnosed and implanted at a gradually earlier age over the last two decades. However, in the large scale study by Geers et al. (2009) on 151 children with CI who were fitted with CI:s before 24 months of age, almost 50% of the children did not have vocabulary skills within the expected range for NH children at age 5–6. Thus vocabulary skills is still an important area of linguistic development for children with CI as it is fundamental both for language abilities in general and for the development of skilled reading.

The educational implications would thus be that the focus of support and teaching in this age group should be both on broadening and deepening of the children’s vocabularies and comprehension of oral language. Of course, early education needs to focus on the decoding aspects of reading but it is also important to consider vocabulary development at an early age. This may be of particular interest as new findings suggest that vocabulary depth may be hard to catch up at later ages (c.f. Walker et al., 2019). The findings from the current research are of clinical importance as delays in spoken vocabulary in children with CI have been reported in a number of studies (Geers et al., 2009; Fagan and Pisoni, 2010; Stiles et al., 2012; Coppens et al., 2013).

Group comparisons and correlation analyses demonstrated that parental education had a significant effect on both word reading and reading comprehension in our sample. The children whose parents had a university degree had significantly higher scores on reading comprehension than children whose parents had high school level education or less. When entered as a predictor variable in the hierarchical regression analysis, parental education did not contribute significantly to reading comprehension. The strong correlation between parental education and receptive vocabulary may, however, suggest that parental education has an indirect effect on reading comprehension through its effect on receptive vocabulary.

Effects of maternal education on children’s language and reading ability have indeed been found in a number of studies on both children with typical hearing (Dollaghan et al., 1999; Magnusson, 2007) and on children with hearing loss (Lieu et al., 2010; Yoshinaga-Itano et al., 2010). The results regarding parental education in the current study should, however, be interpreted with caution as the number of participants in the two groups differed substantially.

Neither complex working memory nor phonological skills contributed significantly to reading comprehension in our group of children with CI. This is not surprising considering the fact that not even decoding was a strong predictor of reading comprehension and that our sample was relatively small.

Regarding the representativeness of the current sample, as noted in Wass et al. (2019), the participants of this study were all recruited from Karolinska University Hospital in Sweden which has a catchment area of around 5 million people and the majority of children implanted in Sweden receive their implants and are followed up regularly by the CI-team at this hospital. The inclusion criterion was that the children should follow the national school curriculum. We thus consider the sample to be relatively representative of children with CI in Sweden who have no additional disabilities that prevent them from attending general education.

Six of the children were reported to use or have used some combination of oral language and sign as support or sign language. This language exposure may thus potentially have had a negative effect on their development of vocabulary and reading comprehension (c.f. Fitzpatrick et al., 2016; Geers et al., 2017). However, as most of the children in the current sample mainly used oral communication, we believe that early exposure to sign as support or sign language are unlikely to affect the current results at a group level.

In summary, it seems that receptive vocabulary is a strong predictor of reading comprehension in 11–12-year-old children with CI. These results support and extend the findings from other studies (Verhoeven and Van Leeuwe, 2008; Ouellette and Beers, 2010; Olson et al., 2011; Melby-Lervåg and Lervåg, 2014; Perfetti and Stafura, 2014) by suggesting that vocabulary is a main predictor of reading comprehension also in children with CI at this age.

## Data Availability Statement

The datasets generated for this study are available on request to the corresponding author.

## Ethics Statement

This study was carried out in accordance with the recommendations of Etikprövningslagen, the Research Ethics Review Committee at Linköping University with written informed consent from all subjects in accordance with the Declaration of Helsinki. The protocol was approved by the Research Ethics Review Committee at Linköping University (Dnr 2011/295-31).

## Author Contributions

MW, LA, BL, EÖ, EK, and UL contributed to the planning and writing phases of the study. UL, EÖ, and LA collected the data. MW drafted the manuscript. LA, BL, EÖ, EK, and UL contributed to the reading and commenting on the drafted manuscript.

## Conflict of Interest

The authors declare that the research was conducted in the absence of any commercial or financial relationships that could be construed as a potential conflict of interest.
